# P-coumaric acid regulates exon 12 splicing of the *ATP7B* gene by modulating hnRNP A1 protein expressions

**DOI:** 10.7603/s40681-015-0010-0

**Published:** 2015-06-06

**Authors:** Ying-Ju Lin, Tsung-Jung Ho, Ting-Hsu Lin, Wei-Yi Hsu, Shao-Mei Huang, Chiu-Chu Liao, Chih-Ho Lai, Xiang Liu, Hsinyi Tsang, Chien-Chen Lai, Fuu-Jen Tsai

**Affiliations:** 1 Genetic Center, Department of Medical Research, China Medical University Hospital, No. 2, Yuh-Der Road, 404 Taichung, Taiwan; 2 School of Chinese Medicine, China Medical University, 404 Taichung, Taiwan; 3 Division of Chinese Medicine, China Medical University Beigang Hospital, 651 Yunlin County, Taiwan; 4 Division of Chinese Medicine, Tainan Manicipal An-Nan Hospital -China Medical University, 709 Tainan, Taiwan; 5 Department of Microbiology, School of Medicine, China Medical University, 404 Taichung, Taiwan; 6 National Institute of Allergy and Infectious Diseases, National Institutes of Health, Bethesda, 20892 Maryland, USA; 7 Institute of Molecular Biology, National Chung Hsing University, 402 Taichung, Taiwan; 8 Department of Biotechnology, Asia University, 413 Taichung, Taiwan

**Keywords:** Wilson’s disease, *Schizonepeta*, *p-coumaric acid*, hnRNP A1, Alternative splicing

## Abstract

*Background:* Wilson’s disease (WD) is a genetic disorder involving the metabolism of copper. WD patients exhibit a wide range of disease phenotypes, including Kayser-Fleischer rings in the cornea, predominant progressive hepatic disease, neurological diseases, and/or psychiatric illnesses, among others. Patients with exon12 mutations of the *ATP7B* gene have progressive hepatic disease. An *ATP7B* gene that lacks exon12 retains 80% of its copper transport activities, suggesting that alternative splicing of *ATP7B* gene may provide alternative therapeutic ways for patients with inherited sequence variants and mutations of this gene. Purpose: We aimed to search for possible Chinese herbs and related compounds for modulating *ATP7B* premRNA splicing.

*Methods:* We used an *ATP7B* exon11-12-13 mini-gene vector as a model and screened 18 Chinese herbal extracts and four compounds from *Schizonepeta* to determine their effects on *ATP7B* pre-mRNA splicing *in vitro*.

*Results:* We found that Schizonepeta demonstrated the greatest potential for alternative splicing activity. Specifically, we found that p-coumaric acid from this herb enhanced *ATP7B* exon12 exclusion through the down-regulation of heterogeneous ribonucleoprotein (hnRNP) A1 protein expressions.

*Conclusion:* These results suggest that there are herbs or herb-related compounds that could modify the alternative splicing of the *ATP7B* gene via a mechanism that regulates pre-mRNA splicing.

## 1. Introduction

Wilson’s disease (WD; MIM number: 277900) is a genetic disorder involving the metabolism of copper [1, 2]. The incidence of the disease is approximately 1 in 30,000 worldwide. It is characterized by copper accumulations, particularly in the liver, kidney, brain, and cornea, resulting from the impairment of the incorporation of copper into ceruloplasmin, with the subsequent excretion of copper through the bile. Patients with WD exhibit a wide range of disease phenotypes, including Kayser-Fleischer rings in the cornea, predominant progressive hepatic disease, neurological diseases, and/or psychiatric illnesses, among others [3]. In addition these phenotypes can present as early as 3 years of age up to as late as the seventh decade of a person’s life. The clinical treatment for WD is copper-chelating agents for the removal of excess copper [4, 5].

The gene responsible for WD is the ATPase *Cu*++ *transporting beta polypeptide* (*ATP7B*, MIM number: 606882). *ATP7B* encodes a copper transporting P-type ATPase, a protein of approximately 160 kDa that contains eight membrane-spanning domains, an ATPase consensus sequence, a hinge domain, a phosphorylation domain, and six copper-binding domains [6]. Over 300 sequence variants and mutations have been identified and collected in the WD Mutation Database to date [1]. We previously observed that predominant mutation hotspots are located in exons 8 and 12 In Taiwanese WD patients [7]. Mutations (Arg919Gly, Thr935Met, Gly943Asp, and 2810delT) in the Transmembrane 6 region (exon12) accounted for 9.62% of all mutations observed in Taiwanese WD patients. These patients with exon12 mutations have progressive hepatic disease [3]. Functional characterization of *ATP7B* showed that variants lacking exon12 retained 80% of their copper transport activities [7], suggesting that the alternative splicing of *ATP7B* gene may provide alternative therapeutic ways for patients with inherited sequence variants and mutations of this gene.

Alternative splicing of mRNA precursors requires specific sequences and splicing factors. Exons frequently contain positive elements known as exonic splicing enhancers (ESEs), which are most often recognized by the serine/arginine-rich family of splicing factors (SR proteins) [8]. Furthermore, exons may also contain splicing silencer elements (ESSs), which are recognized by heterogeneous ribonucleoproteins (hnRNPs) [9]. The presence of ESEs and/or ESSs along with splicing factors regulates alternative splicing. One example of a disease model for alternative splicing that is modulated by SR and hnRNP proteins is spinal muscular atrophy (SMA) disease [10, 11]. This screening model, which prevents exon7 exclusion in the *SMN2* gene, has been widely used, and several pharmacological compounds have been investigated for their splicing correction abilities in order to maintain the full length of SMN2 proteins for therapeutic strategies of SMA disease [12-14].

In the present study, we screened 18 Chinese herbal extracts and four compounds from *Schizonepeta* to determine their effects on *ATP7B* pre-mRNA splicing *in vitro*. We used an *ATP7B* exon11-12-13 mini-gene vector as a model to search for the Chinese herb candidates that modulated alternative *ATP7B* premRNA splicing. This model was based on an *ATP7B* exon11- 12-13 mini-gene and its alternative splicing products [7]. The mini-gene contains three exons and two introns and produces two major alternatively spliced isoforms when expressed in cells. The inclusion form (+ex12) of exon12 produces *ATP7B* mRNA for activating copper-transporting P-type adenosine triphosphatase (ATPase) activities. The exclusion form (-ex12) lacks exon12 of the mini-gene and results in reduced copper transporting ATPase activity, but retains 80% of its biological activities [7]. Exon12 exclusion represents an interesting target due to the mutation hot spots in these regions in Taiwanese WD patients [7, 15]. These patients with exon12 mutations have progressive hepatic disease [3]. In addition, an alternative splice variant of *ATP7B* lacking exon12 was observed in one patient who had a homozygous 2810delT mutation (located in exon12), and showed very mild clinical symptoms [7]. Therefore, it would be useful to identify possible candidate herbs or herb-related compounds that could modify alternative splicing patterns of the *ATP7B* gene. This strategy would be of benefit for alternative splicing therapy for the hundreds of different mutations associated with WD.

## 2. Materials and methods

### 2.1. Plasmids

**Fig. 1:**
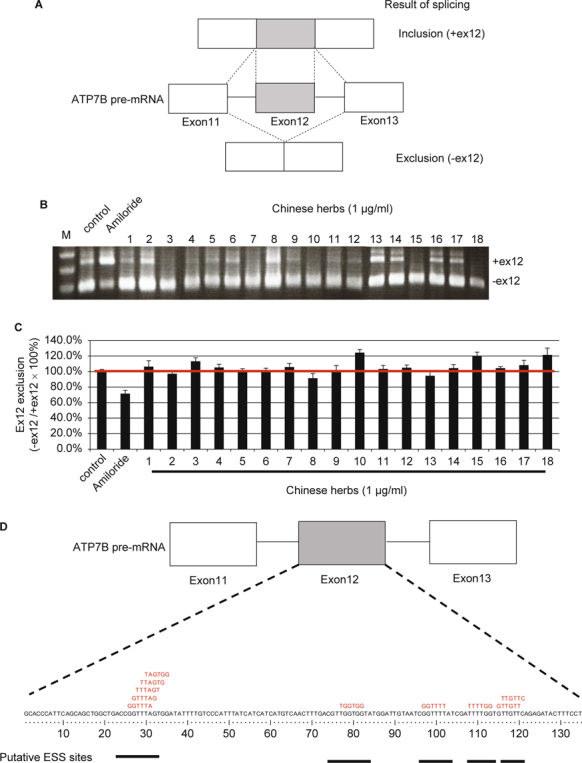
*ATP7B* exon12 exclusion pattern of Chinese herbal extracts: *ATP7B* exon11-12-13 mini-gene vector. (A) Schematic representations of *ATP7B* exon11-12-13 mini-gene vector and its two alternative splicing products. The *ATP7B* mini-gene vector has three exons and two introns and produces two major alternatively spliced isoforms when expressed in cells. (B) RT-PCR analysis of various types of Chinese herbal extracts or Amiloride on *ATP7B* exon11-12-13 mini-gene splicing. CHO-K1 cells were transfected with the *ATP7B* exon11-12-13 mini-gene, held for 24 h, and then treated with various kinds of herbal extracts (1 μg/mL) for another 24 h. Cells were harvested, total RNAs were extracted, and *ATP7B* splicing products were analyzed by RT-PCR. Controls are cells transfected with the *ATP7B* exon 11-12-13 mini-gene vector without any herbs or drugs treatments. Amiloride-treated cells were used for the inhibition of ex12 exclusion controls. (C) Ex12 exclusion ratio analysis of various kinds of Chinese herbal extracts or Amiloride on *ATP7B* exon11-12-13 mini-gene splicing. Quantification was carried out using SynGene Gene Tools. The ex12 exclusion ratio was expressed as: (PCR product (-ex12))/(PCR product (-ex12) + PCR product (+ex12)). Data are expressed as ex12 exclusion ratios (Cell with medium only = 100%) from three independent experiments, each performed in triplicate. (D) Putative ESS sites for hnRNP A1 protein binding.

The *ATP7B* exon11-12-13 mini-gene plasmid has been described previously [7] (Fig. 1A). Briefly, the *ATP7B* (from exon11 to exon13, total 4.5 kb) mini-gene construct was polymerase chain reaction (PCR)-amplified from control genomic DNA by using the forward primer (5 - GTGAGATGGCTTGTTTCATGT-3) and the reverse primer (5 - AACCCAGTGCAGGGCTCACAC-3). The PCR-amplified fragment was cloned into a pcDNA^TM^ 3.1 (Invitrogen) vector, and this fragment was verified by DNA sequencing.

### 2.2. Mammalian cell transfection

CHO-K1 cells were grown in a 6-well plate, and 2.5 μg of the *ATP7B* exon11-12-13 mini-gene plasmid was transiently transfected using Lipofectamine 2000 (Invitrogen). The transfected cells were harvested using trypsin 24 h after transfection, and RNAs were isolated using the RNeasy Mini Kit (QIAGEN).

### 2.3. cDNA synthesis and PCR

Total RNAs from the transfected cells were used for reversetranscription (RT)-PCR using M-MLV reverse transcriptase (Invitrogen), according to manufacturer guidelines. One μL of cDNA was amplified using the forward primer in exon11 (5 - GAGAAGCCATGCCAGTCACTA-3) and the reverse primer in exon13 (5 - CTTGTGCGCCATCTCCAGG -3). Band intensities were quantified using ImageJ software.

### 2.4. Preparation of herbal extracts

Crude extract powders from 18 herbs (Fig. 1) used in traditional Chinese medical practices were obtained from Timing Pharmaceutical, a good manufacturing process (GMP)-certified traditional Chinese medicine manufacturer based in Taiwan. Briefly, each fine herb powder was prepared by milling dry herbal plants with a mechanical grinder and filtering it through a 20-mesh metal sieve, followed by mixing 1.0 g of the powder with 40 mL of distilled water. After boiling for 40 min, the mixture was filtered through a 100-mesh metal sieve. The filtrate (crude water extract) was sterilized using a 0.44 μm syringe filter.

### 2.5. Chemicals

**Fig. 2:**
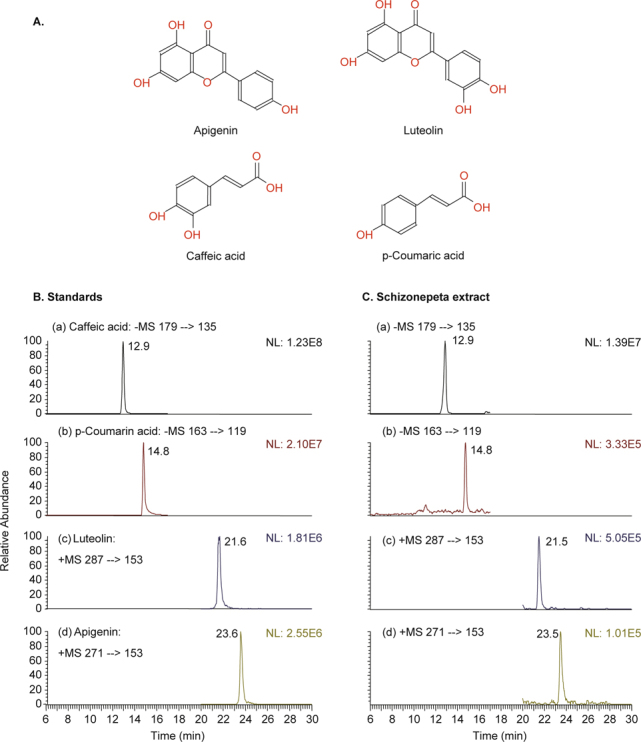
A. Chemical structures of four *Schizonepeta* constituents. Typical multiple reaction monitoring (SRM) chromatograms of of B. Standards: (a) Caffeic acid; (b) p-Coumarin acid; (c) Luteolin; (d) Apigenin; and C. Schizonepeta extract.

Apigenin, luteolin, caffeic acid, and p-coumaric acid were purchased from Sigma Chemical (St. Louis, MO, USA) (Fig. 2). The chemical structures of these compounds are shown in Fig. 2A.

### 2.6. High-performance liquid chromatography with tandem mass spectrometry (HPLC/MS/MS) analyses

HPLC was used to determine the relative abundances of apigenin, luteolin, caffeic acid, and p-coumaric acid in *Schizonepeta* extract. Chromatography was performed using a Finnigan Surveyor ™ HPLC system. HPLC analysis was performed on a 3 μm C18 column (Waters, Atlantis 2.1 mm i.d. × 100 mm). A guard column (Waters, Atlantis , 2.1 mm i.d. × 10 mm) was used to prolong the HPLC column life. The two mobile phases were buffer A, H_2_O (0.1% FA) and buffer B, ACN (0.1% FA). The flow rate was 0.20 mL/min. The gradient conditions were as follows: isocratic elution (75% A) for 5 min, followed by an 8 min gradient to 70% B and then a 12 min gradient to 95% B. Analyses generally lasted for 25 min; an additional 10 min were required for column reequilibration. We used a Finnigan Surveyor™ autosampler fitted with a 2 μL sample loop. HPLC and autosampler systems were synchronized using Xcalibur software.

### 2.7. Antibodies and reagents

Anti-SRp20 (Abcam ab125124), anti-SRSF1 (Abcam ab38017, Taipei, Taiwan), and anti-hnRNP A1 (Abcam ab137780, Taipei, Taiwan) antibodies were purchased from Abcam Taipei, Taiwan. The anti-beta tubulin antibody was obtained from GeneTex (GeneTex GTX101279, Taipei, Taiwan).

### 2.8. Western blot analysis

CHO-K1 cells were added to media containing various chemicals (50 μM) and incubated for 24 h at 37°C. Cells were harvested, washed, and lysed in a lysis buffer (50 mM Tris-HCl [pH 7.5], 150 mM NaCl, 5 mM EDTA, 1% Triton X-100, 0.1% sodium dodecyl sulfate (SDS)) that was supplemented with a protease inhibitor cocktail (Roche). The lysates were resolved by 12% SDSpolyacrylamide gel electrophoresis (PAGE) and transferred to polyvinylidene fluoride membranes (Millipore). The membrane was incubated with primary antibodies overnight at 4°C, and then incubated with alkaline phosphatase-conjugated secondary antibodies (Sigma-Aldrich). Signals were visualized using chemiluminescence following the manufacturer’s protocol (Chemicon).

**Fig. 3:**
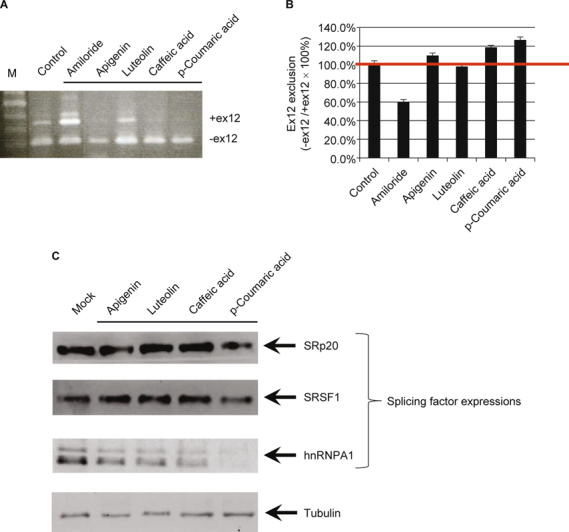
*ATP7B* exon12 exclusion pattern of compounds from *Schizonepeta*: *ATP7B* exon11-12-13 mini-gene vector. (A) RTPCR analysis of various kinds of compounds on *ATP7B* exon11-12-13 mini-gene splicing. CHO-K1 cells were transfected with the *ATP7B* exon11-12-13 mini-gene vector, held for 24 h, and then treated with various kinds of compounds for another 24 h. Cells were harvested, total RNAs were extracted, and *ATP7B* splicing products were analyzed by RT-PCR. Controls are cells transfected with the *ATP7B* exon11-12-13 mini-gene vector without any compound treatment. Amiloride-treated cells were used for the inhibition of ex12 exclusion controls. (B) Ex12 exclusion ratio analysis of various kinds of compounds or Amiloride on *ATP7B* exon11-12-13 mini-gene splicing. Quantification was carried out using SynGene Gene Tools. The ex12 exclusion ratio was expressed as: (PCR product (-ex12))/(PCR product (-ex12) + PCR product (+ex12)). Data are expressed as previously described. (C) Western blot analysis of SRp20, SRSF1, and hnRNP A1 protein expression levels modulated by compounds derived from *Schizonepeta*. CHO-K1 cells were respectively treated with apigenin, luteolin, caffeic acid, and p-coumaric acid for 24 h at 37°C. Cells were harvested, washed, and lysed. The lysates were resolved by 12% SDS-PAGE and western blotting. Briefly, the membrane was incubated with primary antibodies, and then incubated with alkaline phosphatase-conjugated secondary antibodies (Sigma-Aldrich). Signals were visualized using chemiluminescence following the manufacturer’s protocol (Chemicon).

### 2.9. Short interfering RNAs (siRNAs)

siRNAs targeting transcripts for *SRp20* (siSRp20: CCGAAGUGUGUGGGUUGCUAGAAAC; GUUUCUAGCAACCCACACACUUCGG), *SRSF1* (siSRSF1: UGGUGUCGUGGAGUUUGUACGGAAA; UUUCCGUACAAACUCCACGACACCA), and *HNRNP A1* (sihnRNP A1: GGTAGGCTGGCAGATACGTTCGTCA; TGACGAACGTATCTGCCAGCCTACC) were purchased from Invitrogen, Taipei, Taiwan, as was the non-targeting siRNA control (siNC).

### 2.10. siRNA transfection

**Fig. 4:**
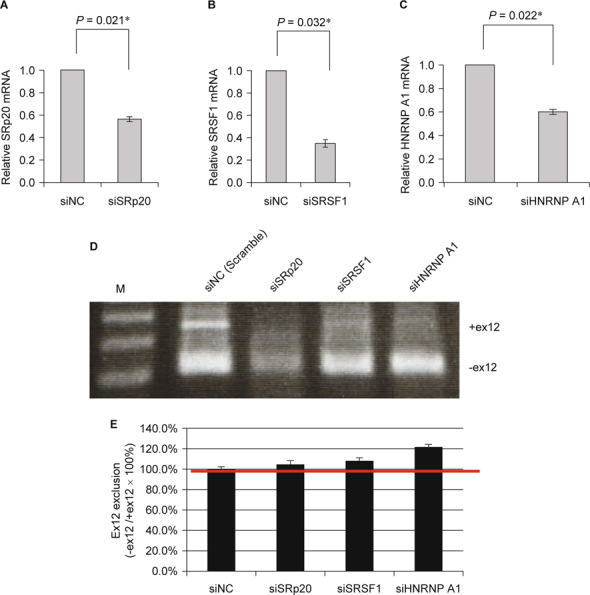
RT-PCR analysis of *ATP7B* exon12 exclusion by siRNAs targeting *SRp20* (siSRp20), *SRSF1* (siSRSF1), and *hnRNP A1* in CHO-K1 cells. (A) RT-qPCR analysis of relative *SRp20* mRNA expression levels. (B) RT-qPCR analysis of relative *SRSF1* mRNA expression levels. (C) RT-qPCR analysis of relative *hnRNP A1* mRNA expression levels. (D) RT-PCR analysis of various kinds of splicing factors on *ATP7B* exon11-12-13 mini-gene splicing using RNA interference. Controls are cells co-transfected with siNC and the *ATP7B* exon11-12-13 mini-gene. (E) Ex12 exclusion ratio analysis of various kinds of splicing factors on *ATP7B* exon11-12-13 mini-gene splicing. Quantification was carried out using SynGene Gene Tools. The ex12 exclusion ratio was expressed as previously described.

CHO-K1 cells (2 × 10^5^) were seeded in 24-well plates and cultured for 24 h. siRNAs targeting *SRp20* (NM_003017), *SRSF1* (NM_006924.4), and *hnRNP* A1 (NM_031157.2) (Fig. 6A, B, and C) were transfected into CHO-K1 cells using Amaxa Nucleofection technology (Lonza) following the manufacturer’s recommendations. Cells were then recovered and seeded in pre-warmed 24-well plates. After 24 h incubation, cells were used for *ATP7B* exon12 exclusion analysis (Fig. 4).

### 2.11. cDNA plasmid transfection

For cDNA plasmid transfection assays, CHO-K1 cells (2 × 10^5^) were seeded in 24-well plates and cultured for 24 h. Cells were transiently transfected with the wild-type expression plasmids, pSRp20, pSRSF1, and phnRNP A1, or with the pcDNA3.0 expression plasmid as a mock control using Amaxa Nucleofection technology (Lonza) following the manufacturer’s recommendations. Cells were then recovered and seeded in pre-warmed 24- well plates. After 24 h incubation, cells were used for *ATP7B* exon12 exclusion analysis (Fig. 5).

## 3. Results and discussion

CHO-K1 cells were transfected with the ATP7B exon11-12-13 mini-gene. Twenty-four hours later, various kinds of herbal extracts (1 μg/mL) were added to the cells (Fig. 1). Cells were harvested, total RNAs were extracted, and *ATP7B* splicing products were analyzed by RT-PCR. The molar ratio of each isoform was analyzed using SynGene Gene Tools. Three of the Chinese herbs evaluated exhibited the most significant splicing efficiency: over 120% compared to untreated cells. Among these three herbs, cells treated with *Schizonepeta tenuifolia* Briq. exhibited the most efficient exon12 exclusion activity at 124.1%. This result suggests that the Chinese herbs *Schizonepeta tenuifolia* Briq., JYH GAN TASO TANG, and CHI BAO MEEI RAN DAN have the ability to modulate alternative splicing through the enhancement of exon12 exclusion of the *ATP7B* gene (Fig. 1).

**Figure:**
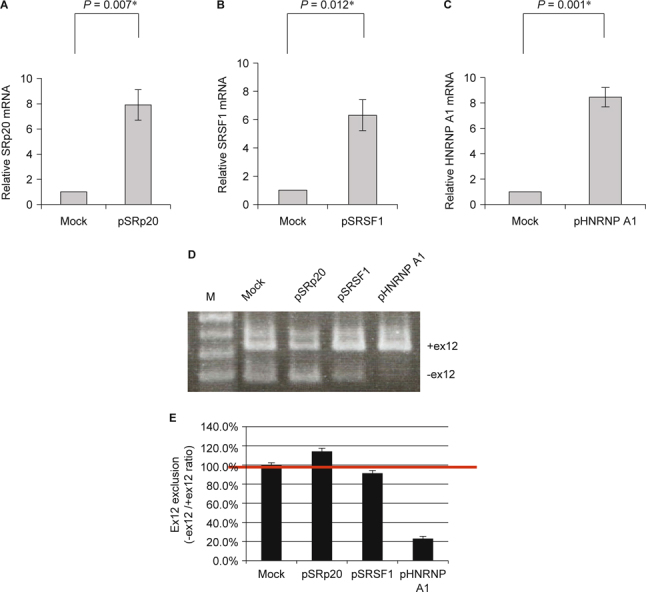
Fig. 5 - RT-PCR analysis of *ATP7B* exon12 exclusion by over-expression of mammalian expression constructs encoding SRp20, SRSF1, and hnRNP A1 in CHO-K1 cells. (A) RT-qPCR analysis of relative *SRp20* mRNA expression levels. (B) RT-qPCR analysis of relative SRSF1 mRNA expression levels. (C) RT-qPCR analysis of relative HNRNP A1 mRNA expression levels. (D) RT-PCR analysis of various kinds of splicing factors on *ATP7B* exon11-12-13 mini-gene splicing by using over-expression of mammalian expression constructs encoding SRp20, SRSF1, and hnRNP A1. Controls are cells co-transfected with pcDNA3.1 vector only and the *ATP7B* exon11-12-13 mini-gene. (E) Ex12 exclusion ratio analysis of various kinds of splicing factors on *ATP7B* exon11-12-13 mini-gene splicing. Quantification was carried out using SynGene Gene Tools. The ex12 exclusion ratio was expressed as previously described.

Among these three herbs, *Schizonepeta tenuifolia* Briq. is the only herbal extract that originates from only one type of plant resource. Derived from the dried aerial parts of *Schizonepeta tenuifolia* Briq. (a member of the Labiatae family of plants), this extract is widely used in Asian countries as an herbal constituent included in treatments for the common cold with fever, ostitis media, skin inflammations, as well as anti-pruritic, anti-microbial, and hemostatic activities [16, 17]. It is categorized as a surfacerelieving agent in modern Chinese Materia Medica guides. Several studies have suggested that *Schizonepeta tenuifolia* Briq. displays immunomodulatory, anti-inflammatory, and anti-oxidant effects. This study is the first to show that the water extract of *Schizonepeta tenuifolia* Briq. also exhibits the ability to modulate alternative splicing via the enhancement of exon12 exclusion of the *ATP7B* gene.

Similar strategies have been widely used in treatments for spinal muscular atrophy (SMA) disease. SMA is caused by the deletion of or mutations in the *SMN1* gene but with the retention of the *SMN2* gene [18]. However, the *SMN2* gene is unable to produce sufficient amounts of SMN proteins for the survival of motor neurons. This is due to the fact that the *SMN2* mRNA transcripts lack exon7, which results in a defective SMN protein [19]. SMA treatment strategies targeting *SMN2* have been proposed [12]; however, unlike the WD strategy, the SMA strategy is aimed at identifying compounds that can enhance the inclusion of exon7 in *SMN2* [12].

Results from HPLC analyses indicated the presence of apigenin, luteolin, caffeic acid, and p-coumaric acid in our *Schizonepeta* water extract (Fig. 2). The HPLC-MS/MS analysis results for four *Schizonepeta*-associated compounds are shown in Fig. 2. HPLC-MS/MS for apigenin (23.6 min retention time), luteolin (21.6 min retention time), cinnamic acid (18.7 min retention time), and p-coumaric acid (14.8 min retention time) from *Schizonepeta* water extract are shown in Fig. 2C. Combined, these results indicated the presence of apigenin, luteolin, caffeic acid and p-coumaric acid in our *Schizonepeta* water extract.

In order to search for chemical compounds from *Schizonepeta* that could modulate alternative *ATP7B* pre-mRNA splicing, CHOK1 cells transfected with the *ATP7B* exon11-12-13 mini-gene were treated with apigenin, luteolin, caffeic acid, and p-coumaric acid, respectively (Fig. 3A and B). Cells were harvested, total RNAs were extracted, and *ATP7B* splicing products were analyzed by RT-PCR. As shown, two compounds, caffeic acid and pcoumaric acid, exhibited the most significant splicing efficiency. Several pharmacological compounds have been investigated and found to modulate the inclusion of exon7 in *SMN2* mRNA. These compounds include aclarubicin (anthracycline antibiotics) [13], benzamide M344 (a histone deacetylase (HDAC) inhibitor) [20], indoprofen (a nonsteroidal anti-inflammatory drug and cyclooxygenase inhibitor) [21], sodium vanadate (a phosphatase inhibitor) [12], 5-(N-ethyl-N-isopropyl)-amiloride (an Na^+^/H^+^ exchanger inhibitor) [14], sodium butyrate (an HDAC inhibitor) [22], and valproic acid (an HDAC inhibitor) [23]. We found that caffeic acid and p-coumaric acid could both enhance exon12 exclusion from the *ATP7B* gene. Caffeic acid and p-coumaric acid are HDAC inhibitors [24]. The HDAC inhibitors, such as benzamide M344, and VPA, also present the ability to correct the splicing abnormality in SMA via regulating splicing factor expression patterns [20, 23]. The present study is the first to demonstrate that caffeic acid and p-coumaric acid can enhance exon12 exclusion from the *ATP7B* gene.

Several studies have suggested that the alternative splicing is modulated by SR and hnRNP proteins in some disease models, including SMA [10, 11]. Furthermore, some HDAC inhibitors, including benzamide M344, and VPA, could also correct the splicing abnormality in SMA, mainly through the regulation of splicing factors [20, 23]. An alternative splicing pattern related to RNA processing machinery was also observed in a mouse model of WD, suggesting that copper elevation selectively alters the protein machinery involved in RNA biogenesis, which is associated with a distinct modification of the splicing pattern [25]. To further explore the mechanism underlying the effect of compounds derived from *Schizonepeta* on *ATP7B* pre-mRNA splicing, CHOK1 cells were respectively treated with apigenin, luteolin, caffeic acid, and p-coumaric acid (Fig. 3). The change in the levels of several SR and hnRNP proteins was examined by western blotting. The results showed that p-coumaric acid markedly decreased the levels of hnRNP A1. These results suggest that p-coumaric acid may modulate *ATP7B* exon12 exclusion through down-regulation of hnRNP A1 protein expressions.

The hnRNP A1 protein belongs to the A/B subfamily of ubiquitously expressed hnRNPs [26]. These proteins are RNA-binding proteins; they form a complex with heterogeneous nuclear RNA (hnRNA); and they modulate pre-mRNA splicing. ESSs are cis-regulatory elements that inhibit the use of adjacent splice sites and prevent exon exclusions [27]. hnRNPs typically function at ESSs and regulate the alternative splicing process. Interestingly, the progression of several diseases is also affected by hnRNP A1- dependent ESS interactions, including Alzheimer’s disease, breast cancer, and SMA [28-30]. Using the ESS prediction website (http://genes.mit.edu/fas-ess/), we observed several putative ESS sites in exon12 of *ATP7B* (Fig. 1D) [27]. These putative ESSs are potential targets for hnRNP A1 protein binding to prevent *ATP7B* exon12 exclusion.

To address whether or not *ATP7B* exon12 exclusion can be modulated by these splicing factors, *in vivo* splicing assays were performed in CHO-K1 cells using RNA interference experiments (Fig. 4). CHO-K1 cells were transiently co-transfected with siRNAs targeting SRp20 (siSRp20), SRSF1 (siSRSF1), and hnRNP A1 (sihnRNP A1), respectively, along with the *ATP7B* exon11- 12-13 mini-gene, and the effect was assessed by real-time quantitative PCR (qPCR) (Fig. 4 A, B, and C). Cells were harvested, total RNAs were extracted, and *ATP7B* splicing products were analyzed by RT-PCR. As shown in Fig. 4D, compared to the negative control siRNA (siNC), transfection with sihnRNP A1 exhibited the most significant splicing efficiency. This result suggests that down-regulation of the hnRNP A1 protein is able to modulate alternative splicing by the enhancement of exon12 exclusion of the *ATP7B* gene (Fig. 4 D and E).

To further address whether or not *ATP7B* exon12 exclusion can be modulated by these splicing factors, *in vivo* splicing assays were also performed in CHO-K1 cells by overexpression experiments using mammalian expression constructs encoding SRp20, SRSF1, and hnRNP A1 (Fig. 5). The effect was assessed by real-time qPCR (Fig. 5 A, B and C). The CHO-K1 cells were co-transfected with these expression constructs and the *ATP7B* exon11-12-13 mini-gene. Cells were harvested, total RNAs were extracted, and *ATP7B* splicing products were analyzed by RTPCR. As shown in Fig. 5D, compared to the control pcDNA3.1 vector only, transfection with phnRNP A1 exhibited the most significant splicing efficiency. This result suggests that overexpression of the hnRNP A1 protein can modulate alternative splicing by the inhibition of exon12 exclusion of the *ATP7B* gene (Fig. 5 D and E).

In this study, we first found that the HDAC inhibitor, pcoumaric acid, from *Schizonepeta* water extract could inhibit the expression of the splicing factor, hnRNP A1. There are several putative ESS sites found in exon12 for hnRNP A1 protein binding. RNA interference of hnRNP A1 promoted *ATP7B* exon12 exclusion. In addition, over-expression of the hnRNP A1 protein promoted *ATP7B* exon12 inclusion. The protein patterns of host splicing proteins may be altered when cells are treated with pcoumaric acid, and induced or inhibited cellular proteins associated with ESS may influence pre-mRNA splicing. Additional studies are required to determine whether these HDAC inhibitors and induced or inhibited ESS binding proteins are essential for alternative splicing or not, and to identify new targets for agent development.

## Competing interests

The authors declare that they have no competing interests.

## Acknowledgments

This research was supported by grants from the China Medical University (CMU102-PH-01), the China Medical University Hospital (DMR-104-029), and the Republic of China National Science Council (NSC101-2314-B-039-008-MY3). We thank Dr. Willy W. L. Hong for technical help and suggestions. We also thank the supports from the China Medical University under the Aim for Top University Plan of the Ministry of Education, Taiwan.
